# Combined sterile insect technique and incompatible insect technique: The first proof-of-concept to suppress *Aedes aegypti* vector populations in semi-rural settings in Thailand

**DOI:** 10.1371/journal.pntd.0007771

**Published:** 2019-10-28

**Authors:** Pattamaporn Kittayapong, Suwannapa Ninphanomchai, Wanitch Limohpasmanee, Chitti Chansang, Uruyakorn Chansang, Piti Mongkalangoon

**Affiliations:** 1 Center of Excellence for Vectors and Vector-Borne Diseases, Faculty of Science, Mahidol University at Salaya, Nakhon Pathom, Thailand; 2 Department of Biology, Faculty of Science, Mahidol University, Bangkok, Thailand; 3 Thailand Institute of Nuclear Technology, Ministry of Science and Technology, Nakhon Nayok, Thailand; 4 Department of Medical Sciences, Ministry of Public Health, Nonthaburi, Thailand; 5 Department of Disease Control, Ministry of Public Health, Nonthaburi, Thailand; University of Hawaii at Manoa, UNITED STATES

## Abstract

**Background:**

Important arboviral diseases, such as dengue, chikungunya, and Zika virus infections, are transmitted mainly by the *Aedes aegypti* vector. So far, controlling this vector species with current tools and strategies has not demonstrated sustainable and significant impacts. Our main objective was to evaluate whether open field release of sterile males, produced from combining the sterile insect technique using radiation with the insect incompatible technique through *Wolbachia*-induced incompatibility (SIT/IIT), could suppress natural populations of *Ae*. *aegypti* in semi-rural village settings in Thailand.

**Methodology/Principal findings:**

Irradiated *Wolbachia*-infected *Aedes aegypti* males produced by the SIT/IIT approach were completely sterile and were able to compete with the wild fertile ones. Open field release of these sterile males was conducted in an ecologically isolated village in Chachoengsao Province, eastern Thailand. House-to-house visit and media reports resulted in community acceptance and public awareness of the technology. During intervention, approximately 100–200 sterile males were released weekly in each household. After 6 months of sterile male release, a significant reduction (*p*<0.05) of the mean egg hatch rate (84%) and the mean number of females per household (97.30%) was achieved in the treatment areas when compared to the control ones.

**Conclusions/Significance:**

Our study represents the first open field release of sterile *Ae*. *aegypti* males developed from a combined SIT/IIT approach. Entomological assessment using ovitraps, adult sticky traps, and portable vacuum aspirators confirmed the success in reducing natural populations of *Ae*. *aegypti* females in treated areas. Public awareness through media resulted in positive support for practical use of this strategy in wider areas. Further study using a systematic randomized trial is needed to determine whether this approach could have a significant impact on the diseases transmitted by *Ae*. *aegypti* vector.

## Author summary

Dengue, chikungunya, and Zika are important diseases that pass to humans only by the bites of *Aedes aegypti* mosquitoes infected with these viruses. Due to the lack of an effective vaccine and drug to cure these diseases, mosquito control is the only method available to reduce disease risk. Most of the available preventive and control measures involve an application of chemical-based products. The inappropriate use of chemicals causes insecticide resistance and environmental contamination. In this study, we report on the pilot trial of an alternative, safe, and environmental friendly approach to suppress *Ae*. *aegypti* mosquito populations. This alternative method involves the release of sterile *Ae*. *aegypti* males that were infected with, naturally-occurring, insect bacteria *Wolbachia*, and were irradiated at low doses to obtain full sterilization. The pilot trial involved the release of 100–200 sterile males per household in a treated area over a 6-month period. High efficacy of this approach was evidenced from a significant reduction in the numbers of wild *Ae*. *aegypti* females and an increase in sterility by reduction in the egg hatch rate of wild *Ae*. *aegypti* females in the treated area. Public awareness through media resulted in public support to conduct trials in wider areas.

## Introduction

Mosquito-borne diseases continuously cause enormous suffering in humans worldwide, both with regards to mortality and morbidity, particularly dengue and malaria [1–4]. In particular, dengue is causing important public health problems and high economic burdens globally [5,6]. According to the World Health Organization (WHO), more than 2,500 million people in over 100 countries are at risk of infection by the dengue virus. According to the WHO regions of southeast Asia, 1.3 billion people, who live in 10 dengue endemic southeast Asian countries, bear major morbidity and economic burdens. The overall estimated annual economic burden of dengue was US$950 million, estimated from the total number of cases and unit cost per dengue episode in southeast Asia. Chikungunya, another viral disease that is transmitted to humans by infected *Aedes* mosquitoes, was originally confined to Africa but has more recently spread rapidly across the Indian Ocean, Europe, and the Americas [7]. Most recently, Zika disease outbreaks in several parts of the world made it necessary to adopt a new and effective methodology to control the *Ae*. *aegypti* vectors causing the disease [8,9].

In the absence of highly effective vaccines and/or efficient, safe, and inexpensive drugs to combat dengue, chikungunya, and Zika, many consider population control of the insect vectors to be the most effective way to manage these diseases [1,10]. Most vector control strategies are insecticide-based and their widespread use resulted in increasing incidences of insecticide resistance [11–13]. Moreover, existing mosquito control tools failed to prevent a global increase in the incidence of mosquito-borne diseases [14], thus there is an urgent need for complementary sustainable and environmental-friendly tools and approaches for vector control. Recently, there has been an increase of interest in the deployment of *Wolbachia* symbionts and the sterile insect technique (SIT) as alternative or complementary tools to battle mosquito vectors.

SIT is a method of insect pest control with a strong record of success against a wide range of agricultural pests. This technique has the potential to be applied to mosquito vectors. Because of the low environmental impact and relatively unobtrusive means of deployment, particularly in urban areas, SIT has been well accepted. The technique consists of releasing a very large number of sterile males in order to mate with native females present in the environment. With a sufficiently high number of sterile male released, the number of native insects decreases over time, thus driving the native populations to local extinction. Sterile insect technique (SIT) has been successfully applied in large-scale operations in many countries to control insect pests and to prevent huge financial losses as a result of lost crops of economic importance [15].

*Wolbachia* is an intracellular bacterium and was first reported in the reproductive tissues of *Culex pipens* in 1924 [16]. Their widespread distribution and various effects on the reproductive system of the hosts places these bacteria among the most popular ones for use in suppressing mosquito vectors of serious vector-borne diseases, i.e., malaria, dengue, and filariasis [17–19]. However, as some important mosquito species, especially *Aedes aegypti* [20] and many *Anopheles* species [21], were not naturally infected with *Wolbachia*, a microinjection technique has been applied for introduction of *Wolbachia* into these mosquito vectors, not only at the embryonic stage [22] but also the adult stage [23]. Recent studies show that *Wolbachia* trans-infected mosquito vectors have had limited infections of arboviruses, for example, dengue [24], chikungunya [24,25], Yellow fever [25], West Nile [26], and Zika [27], as well as malaria parasites [24,28,29].

The objective of our studies was to conduct the pilot trial to suppress *Ae*. *aegypti* mosquito vectors by applying a combination of sterile insect technique (SIT) and insect incompatible technique (IIT) in producing sterile males for open field release. This combined strategy had some benefits over either approach conducted alone. First, double sterilization process assures sterility of the released males, since low-dose radiation could sterilize those that do not contain *Wolbachia*. The system of SIT/IIT is particularly useful when the CI is incomplete due to imperfect maternal transmission; like in our case the CI was reported to be about 50% [23]. Second, sterilization of *Wolbachia*-infected females with radiation should prevent fertility of *Wolbachia*-infected mating pairs in the wild, since sterility of *Wolbachia*-infected females could only be caused by irradiation; and as a consequence, no environmental contamination by permanent replacement of natural mosquito populations with the *Wolbachia*-infected ones could occur. Third, if *Wolbachia*-infected females were accidentally released in the wild, they should be sterile and should not become vectors due to the anti-viral property of the *Wolbachia* bacteria [24].

## Materials and methods

### Development of the Thai *Wolbachia* trans-infected *Aedes aegypti* line

#### *Aedes aegypti* mosquitoes and *Wolbachia* strains

The materials, i.e., *Aedes aegypti* mosquitoes, were originally collected from several villages in Hua Sam Rong Subdistrict, Plaeng Yao District, Chachoengsao Province, eastern Thailand. They were colonized and maintained at the screen climatic control insectary of the Center of Excellence for Vectors and Vector-Borne Disease, Faculty of Science, Mahidol University, Salaya Campus, Nakhon Pathom Province, Thailand, with 75 ± 2% humidity, 27 ± 2°C temperature, and a photoperiod of L12:D12. The donor *Wolbachia* strains were *w*AlbA and *w*AlbB extracted from ovaries of *Ae*. *albopictus* collected in the rubber plantations in the same areas.

#### Extraction of *Wolbachia* from *Ae*. *albopictus*

*Wolbachia* was purified from dissected ovaries of 2 week old *Ae*. *albopictus* females. Ovary dissection was carried out under stereomicroscope using microscissors. Dissected ovaries were homogenized with PBS solution to preserve quality of bacteria and then were centrifuged at 500 rpm for 5 minutes to remove impurities. The supernatant containing live *Wolbachia* was transferred to a new tube and centrifuged at 8,000 rpm for 5 minutes. Later, the supernatant was discarded and the pallet at the bottom of the tube was re-suspended in 30 μl PBS [30].

#### Direct microinjection of *Wolbachia* into *Aedes aegypti*

The solution purified from *Ae*. *albopictus* ovaries was confirmed for the presence of *Wolbachia* prior to transinfection into the *Ae*. *aegypti* recipient using PCR. Purified live *Wolbachia* was microinjected into 3–5 days old unmated *Ae*. *aegypti* female recipients at the thorax region, using an IM300 microinjector (Narishige Scientific; Tokyo, Japan) [23]. After microinjection, *Wolbachia-*transinfected *Ae*. *aegypti* females were put back in cages to let them recover from injuries due to microinjection. After 24 hours, they were fed with 10% sugar solution. Temperature and relative humidity of the insectary were regulated at 27 ± 2°C and at 75 ± 2% respectively. About 3–5 days after microinjection, uninfected males were put in cages to allow mating. A few days after mating, these females were then fed with human blood. Oviposition cups were put in cages 24 hours after blood feeding to collect eggs for establishing the next generation.

#### Detection of *Wolbachia* in trans-infected *Ae*. *aegypti*

After microinjection, 5–10 trans-infected female *Ae*. *aegypti* (F0) were taken from cages to detect for *Wolbachia* after egg-laying. Similarly, their offspring were later checked for *Wolbachia* maternal transmission using PCR [31]. Each generation, randomly selected offspring were detected for *Wolbachia* infection. Mosquitoes were ground in 100 μl STE (100 mM NaCl, 10 mM Tris-HCl, 1 mM EDTA, pH 8.0) at 3,000 rpm for 3–5 minutes using Tissue lyser II, with the aid of 3 mm bead. The grinding solution was heated at 95°C for 10 minutes and then centrifuged at 14,000 rpm for 1 min. *Wolbachia* DNA was amplified in 20 μl mixture of 2 μl of 10x buffers, 1 μl of 50mM MgCl_2_, 1 μl of 10 mM dNTP, 0.5 μl of forward and reverse primers, 1 μl of Taq DNA polymerase (Invitrogen, USA), and 12 μl of distilled water. PCR was carried out to 35 cycles at 95°C for 3 min, followed by 95°C for 1 min, 50°C for 1 min, and 72°C for 1 min. PCR products were electrophoresed on 2% agarose gel in TAE buffer strained with ethidium bromide. DNA bands were visualized under UV light inside a GelDoc machine.

General primers used to detect *Wolbachia* were wsp 81F (5'-TGG TCC AAT AAG TGA AGA AAC- 3’) and wsp 691R (5'- AAA AAT TAA ACG CTA CTC CA-3’) [32]. Mosquitoes that tested positive for *Wolbachia* were further tested by PCR using specific primers: 1) wsp 328F (5′-CCA GCA GAT ACT ATT GCG-3′) and wsp 691R (5' AAA AAT TAA ACG CTA CTC CA*-*3′) for *w*AlbA, 2) wsp 81F (5'-TGG TCC AAT AAG TGA AGA AAC-3′) and wsp 522R (5'-ACC AGC TTT TGC TTG ATA-3′) for *w*AlbB.

### Preparation of sterile *Aedes aegypti* males for pilot trial

#### Sex separation and male sterilization using radiation

*Wolbachia*-infected *Ae*. *aegypti* male and female pupae were filtered and separated by using larval-pupal separators (Model 5412, John W. Hock Company, Gainesville, FL, USA) [33]. After sex separation, 500 male pupae were put in each 12.5-inch round plastic container with screened cover and half filled with clean water. All containers filled with male pupae were then transported to the Thailand Institute of Nuclear technology (TINT) in Nakhon Nayok Province for radiation using a Cobalt-60 (Gammar Chamber 5000, Board of Radiation and Isotope Technology (BRIT), DAE, Mumbai, India) at the dosage of 70 Gy for 45 sec per 500 pupae per container.

A total numbers of 40 sampling males from each irradiation lot were then transported back to the laboratory at the Center of Excellence for Vectors and Vector-Borne Diseases, Faculty of Science, Mahidol University, Salaya Campus, Nakhon Pathom Province. Mating experiments of these irradiated *Wolbachia*-infected males were conducted weekly with the non-irradiated wild type *Ae*. *aegypti* females as mating pairs, according to Kittayapong et al. (2018) [33]. The total number of eggs laid, the total number of hatched eggs, and the egg hatch rate of individual females were recorded. In addition, the infection rate of *Wolbachia* was determined in 40 samples of each irradiation lot.

#### Experiment on male competitiveness of irradiated *Wolbachia*-infected *Aedes aegypti* males

An experiment was conducted to determine the mating competitiveness of irradiated *Wolbachia*-infected *Ae*. *aegypti* males after being irradiated at 70 Gy. The mating ratios between irradiated *Wolbachia*-infected males (♂ir-W) vs non-irradiated wild type males (♂nr-w) and non-irradiated wild type females (♀nr-w) were 1:1:1, 5:1:1, 10:1:1, and 20:1:1 respectively. Each non-irradiated wild type female was put in a separate cage sized 10 cm x 10 cm x 10 cm. and one non-irradiated wild type male and 1, 5, 10, or 20 irradiated *Wolbachia*-infected males were introduced into each cage respectively. The number of replicates for each mating ratio ranged from 12 to 16. These mosquito cages were left in an insectary at 75 ± 2% humidity, 27 ± 2°C temperature, and a photoperiod of L12:D12. A 10% sucrose solution was provided as food source for adult mosquitoes, and pig blood was provided as blood meals for females a few days after male introduction. The oviposition cups were introduced to each mosquito cage to collect eggs. After a few days, each egg paper in the oviposition cup was collected, dried at room temperature, and then the eggs were counted before hatching in deionized water. The total numbers of eggs hatched and the egg hatch rate of each egg batch were recorded to determine the male mating competitiveness. The male mating competitiveness index (C) was calculated as C = [(Hn–Ho)/(Ho–Hs)] *(N/S); where Hn is the hatch rate from eggs of wild females mated with fertile males, Ho is the hatch rate from eggs from each experimental cage, Hs is the hatch rate from eggs of females mated with sterile males, and N and S are the numbers of fertile and sterile males respectively [34,35]. The induce sterility (IS) was assessed in order to evaluate the effect of sterile male releases, and it was calculated as 100% minus the residual fertility value, which was calculated as IS = 100%–(Ho/Hn) [35].

#### Handling and transportation of irradiated *Wolbachia*-infected *Aedes aegypti* males

Irradiated *Wolbachia*-infected male pupae prepared for field release were further transported to the field lab station in Chachoengsao Province and were left overnight at 27°C. The next day after irradiation, male pupae were counted, and 100 of them were put in each delivery plastic container with screened cover and a plastic tube filled with 10% sugar solution as food source. Emerged mosquitoes were sampled and inspected by well-trained and experienced field workers; and if any irradiated *Wolbachia*-infected females were mixed with the males in the delivery containers, they were discarded and replaced with the irradiated *Wolbachia*-infected males. Newly emerged males were left at 27°C overnight in the field lab station for sugar feeding and recovering before being released. Approximately 100–250 delivery containers filled with only males were prepared each week for open field release.

### Preparation of study sites and community/public engagement

#### Description of the pilot study site

**Fig 1:**
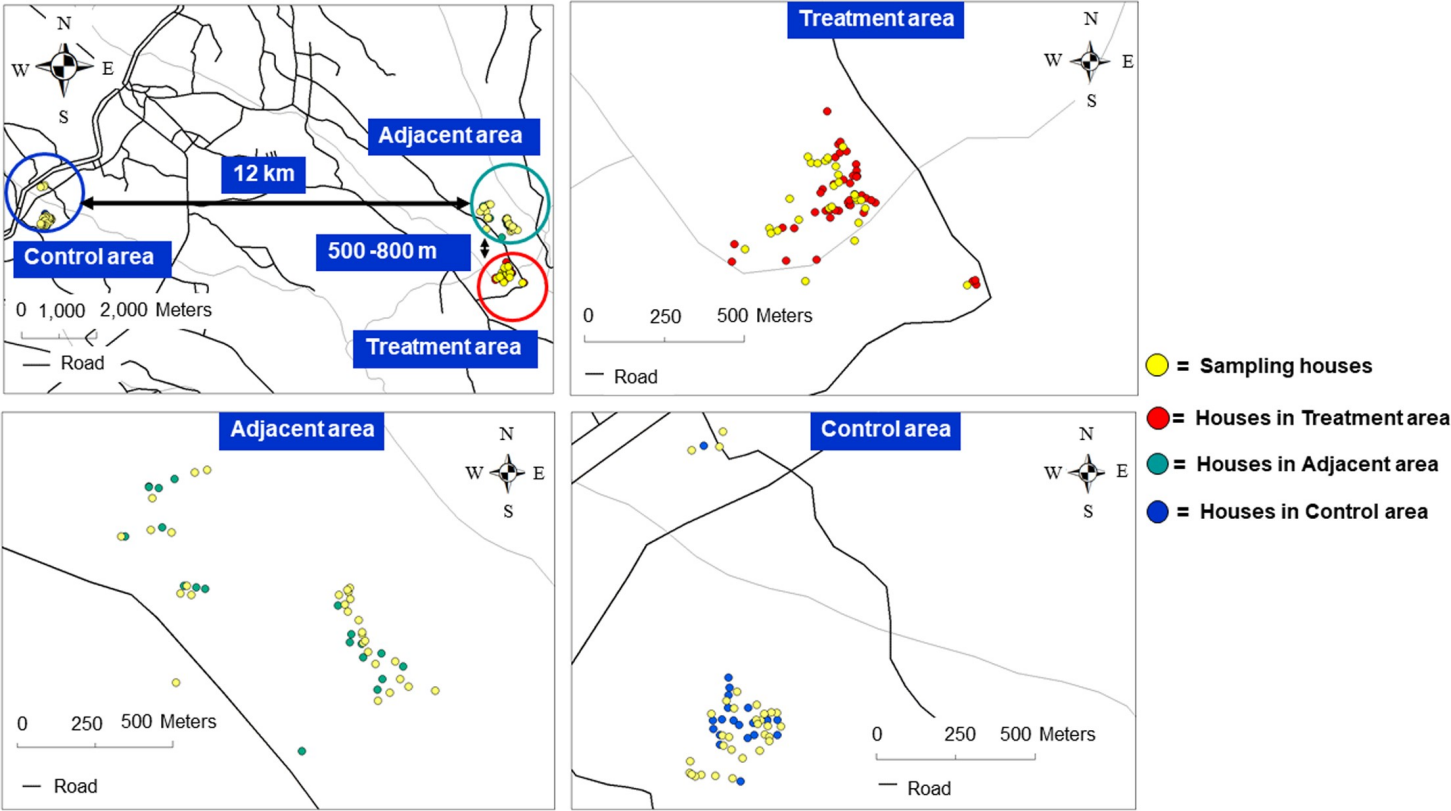
GPS location of households in the treatment, the adjacent and the control areas in Plaeng Yao District, Chachoengsao Province, eastern Thailand, showing the sampling houses and the distance among them.

The study site was located in Plaeng Yao District, Chachoengsao Province, eastern Thailand (13°36'44.91"N, 101°16'29.37"E). Three study areas were selected: Nong Satit as the treatment area (0.65 sq.km.), Pleang Mai Daeng as the adjacent area (1.02 sq.km.), and Nong Sarika as the control area (0.52 sq.km.). The distance between the treatment and the adjacent area, which had the closest cluster of houses, was approximately 500–800 meters; while the treatment area and the control area was approximately 12 kilometers apart (Fig 1). The study areas were located among rice field, cassava, rubber, and other plantations. The populations of the study site were approximately 10,000 inhabitants at the sub-district level.

#### Community/public engagement

Before the intervention, several meetings were held with local governmental authorities, school teachers, and householders in local communities to inform them about the objectives and activities of this pilot trial. Education meetings with live demonstration, particularly on the life cycle and feeding behavior of *Ae*. *aegypti* mosquitoes, were organized in the communities in order to deliver the key message that the male mosquitoes could not bite and they only fed on nectars. In addition, the Ministry of Public Health also provided the information related to sterile mosquitoes with a hot line for questions on their official website.

Public engagement was obtained through media channels. There were a few press releases by Mahidol University and Department of Disease Control of the Ministry of Public Health of Thailand in support of the pilot sterile mosquito project. Other media channels were international and national news, a documentary, and TV shows. Some national news was delivered by relevant institutions, including the Thailand Institute of Nuclear Technology (TINT), Ministry of Science and Technology of Thailand.

To increase public awareness, the pilot project was launched on ASEAN Dengue Day (June 15, 2016) with participation from representatives of 13 different Asian countries. Local communities and local government authorities, including local schools, were involved in the organization of the opening ceremony at the Nong Satit School in the center of the treated village.

### Open field release of sterile males and entomological evaluation

#### Ethics approval

The pilot suppression trial of *Aedes aegypti* using a combined sterile insect technique and *Wolbachia*-based approach in Plaeng Yao District, Chachoengsao Province, eastern Thailand, was reviewed and approved by the Mahidol University Institutional Review Board (MU-CIRB 2016/085.0407).

#### Release of sterile *Aedes aegypti* males

On the day of the open field releases, all delivery containers filled with sterile *Ae*. *aegypti* male mosquitoes were transported to the center of the treated village during the morning hours. Local health volunteers participated in weekly delivery of the plastic containers filled with sterile males to each household in the treatment area; and some homeowners participated in the release of these sterile mosquitoes in their homes. Sterile males were released from individual containers, either inside or outside of each household. At the end of the day, field workers inspected the delivery containers to make sure that all sterile males were released before leaving the study areas. The total numbers of sterile male mosquitoes being released per week were between 10,000 to 25,000 individuals. The release of these sterile males continued weekly for a total of 24 weeks or 6 months (July-December 2016).

#### Entomological monitoring of natural *Aedes aegypti* populations

Natural *Ae*. *aegypti* mosquito populations in the treatment area, the adjacent area. and the control area were monitored by using adult sticky traps (MosHouse) (Go Green Co., Ltd., Nakhon Pathom, Thailand) and portable vacuum aspirators (MosVac) (Go Green Co., Ltd., Nakhon Pathom, Thailand). MosHouse is an adult mosquito trap that receives its name from its external house shape. Mosquitoes were collected through the double sticky panels placed in the middle of the trap. MosVac is a portable vacuuming aspirator equipped with rechargeable batteries. Adult mosquitoes were collected in the screen-covered plastic container at the tip of the device [36]. Well-trained and experienced field staffs supervised and coordinated with local public health volunteers and local field workers for sample collection from sampling households. MosHouse sticky traps were distributed on the first week of the month in 60 sampling houses: 20 traps each in the treatment area, the adjacent area, and the control area. The traps were left for 7 days to allow mosquitoes to get struck on the double sticky panels placed inside. The sticky panels with mosquitoes from the traps were labeled with date and location and were placed in a row in the specific collection boxes before being transported to the field lab station for identification; the number of *Ae*. *aegypti* males and females were then recorded. In addition, the portable vacuum aspirators, MosVac, were used to collect resting mosquitoes in the same 60 households. Sampling mosquitoes were captured alive in the plastic containers of the aspirators. They were then killed in the refrigerator, transferred to plastic vials, and labeled with the date and location before being transported in coolers filled with ice packs to the field lab station for mosquito identification. The total numbers of *Ae*. *aegypti* males and females were monthly recorded in order to monitor field populations over time. Both adult sticky traps and portable aspirators were used once a month from July-December 2016 for a total of 6 months.

During the study period, ovitraps were distributed both inside and outside 60 houses, with a total of 120 ovitraps in the treatment area, the adjacent area, and the control area, for a total of 24 weeks. The filter papers with *Aedes* eggs from ovitraps were collected and replaced weekly. The egg papers were placed in plastic bags, labeled with date and location, before being transported to the entomological laboratory at the National Institute of Health, Department of Medical Sciences, Ministry of Public Health in Nonthaburi Province for egg counting and egg hatching, in order to monitor the egg hatch rate of wild *Ae*. *aegypti* females.

### Follow-up mark-release-recapture of sterile males

After completion of the intervention, a small-scale mark-release-recapture experiment was carried out at the study site to determine the distance that the sterile males could disperse and the number of days that they could live in the field after release. A total number of 1,400 *Wolbachia*-infected *Ae*. *ageypti* males was irradiated at 70 Gy at the pupal stage as previously described. They were marked by being fed on a 1% Rhodamine-B mixed with 10% sucrose solution at the adult stage; and then were released at the center point in Nong Sarika Village of Plaeng Yao District, Chachoengsao Province. The mosquitoes were collected from the sampling households at a radius of 1 km by using MosVac portable vacuum aspirators. The collection was conducted for 30 days: every day during the first week and every other day for the second week to the fourth week. All collected mosquitoes were knocked down by ice, put in labeled plastic tubes, and then transported back to the laboratory for identification of Rhodamine-marked male mosquitoes using a fluorescent stereomicroscope.

## Results

### Establishment of the Thai *Wolbachia*-infected *Aedes aegypti* line

A *Wolbachia* super-infected *Ae*. *aegypti* line (ThAB), containing *Alb*A and *Alb*B strains, was developed by direct microinjection technique, as described in Ruang-areerate and Kittayapong, (2006) [23]. The strain had previously been characterized, and cytoplasmic incompatibility was shown to be incomplete [23]. In addition, survival and longevity were reported in Kittayapong, et al. (2018) [33]. In this study, further characterization was conducted to evaluate the sterility and mating competitiveness after the mosquitoes were irradiated.

### Evaluation of sterility of irradiated *Wolbachia*-infected *Aedes aegypti* males

A total numbers of 960 males were sampled from 24 lots of irradiated *Wolbachia*-infected *Ae*. *aegypti* males that were prepared for weekly open field release, in order to evaluate their sterility as part of the quality control for mass production of sterile males. Overall, the irradiated *Wolbachia*-infected males from all 24 lots showed high sterility with very low egg hatch rates (mean eggs per female = 33.53 ± 10.79, mean hatched eggs = 1.04 ± 2.18, mean egg hatch rate = 0.14 ± 0.27). In addition, fifteen lots (62.5%) showed complete sterility with no hatched eggs (mean eggs per female = 31.00 ± 11.68, mean hatched eggs = 00.00 ± 00.00, mean egg hatch rate = 00.00 ± 00.00) (Table 1). Results from our mating experiments confirmed complete or near complete sterility of the released males, assuring an introduction of sterility into the wild female populations in the field.

**Table 1:** Cross-mating between irradiated *Wolbachia*-infected males and non-radiated wild type females of *Aedes aegypti* mosquitoes, in order to evaluate sterility of each radiation lot as a quality control.

Lot no.	Mating Pairs	Mean no. eggs	Mean no. egg/female	Mean no. hatched eggs	Mean egg hatch rate
1	40	1,208.21	30.21	1	0.08
2	40	1,908.57	47.71	1	0.06
3	40	1,771.43	44.29	2	0.13
4	40	757.65	18.94	0	0.00
5	40	1,594.44	39.86	0	0.00
6	40	921.11	23.03	0	0.00
7	40	1,388.89	34.72	4	0.32
8	40	1,065.00	26.63	0	0.00
9	40	1,029.09	25.73	2	0.35
10	40	1,000.00	25.00	0	0.00
11	40	856.00	21.40	0	0.00
12	40	1,134.55	28.36	0	0.00
13	40	1,040.00	26.00	0	0.00
14	40	980.00	24.50	0	0.00
15	40	1,192.00	29.80	0	0.00
16	40	1,626.00	40.65	2	0.25
17	40	2,512.00	62.80	0	0.00
18	40	1,869.57	46.74	10	0.93
19	40	1,200.00	30.00	0	0.00
20	40	1,600.00	40.00	1	0.15
21	40	1,926.67	48.17	0	0.00
22	40	942.22	23.56	0	0.00
23	40	1,182.86	29.57	2	0.97
24	40	1,480.00	37.00	0	0.00
**24**	**40**	**1,341.13**	**33.53**	**1.04**	**0.14**

In addition, PCR detection of *Wolbachia* in 40 irradiated male mosquitoes, sampling from each irradiation lot, showed that the mean percentage of *Wolbachia* infection was 50.21 ± 0.49%.

### Evaluation of mating competitiveness of irradiated *Wolbachia*-infected *Aedes aegypti* males

Irradiated *Wolbachia*-infected males (♂ir-W) were evaluated for their competiveness with non-radiated wild type males (♂ir-w), in order to mate with non-radiated wild type females (♀nr-w) at different mating ratios (♂ir-W:♂nr-w:♀nr-w). Mating between the fertile males and females (Hn = 0.90), and the sterile males and females (Hs = 0.00) were used as a fertile and a sterile control respectively. At the mating ratios of 1:1:1, 5:1:1, and 10:1:1, hatched eggs were still observed (egg hatch rate _(1:1:1)_ = 0.60 (0.59 ± 0.17); egg hatch rate _(5:1:1)_ = 0.18 (0.18 ± 0.05); egg hatch rate _(10:1:1)_ = 0.14 (0.15 ± 0.08) but a huge reduction in the egg hatch rate was detected at the mating ratios of 20:1:1 (egg hatch rate _(20:1:1)_ = 0.04 (0.04 ± 0.03) (Table 2). Induced Sterility (IS) was compared with the theoretical value of 100 (IS = 100 when the male is completely sterile) by using the one sample *t*-tests (Table 2). IS was significantly increased when compared to the theoretical value at the mating ratios of 1:1:1 (Mean ± 95% CI = 99.34 (-0.73 –-0.59), *t* = -19.393, *df* = 29, *p* = 0.000), 5:1:1 (Mean ± 95% CI = 99.80 (-0.22 –-0.18), *t* = -18.865, *df* = 29, *p* = 0.000), 10:1:1 (Mean ± 95% CI = 99.83 (-0.20 –-0.13), *t* = -10.733, *df* = 29, *p* = 0.000) and 20:1:1 (Mean ± 95% CI = 99.95 (-0.06 –-0.04), *t* = -8.082, *df* = 29, *p* = 0.000). IS was significantly increased when the mating ratios increased, and the highest IS was observed at the mating ratio of 20:1:1. For mating competitiveness index (C), the data compared the C values with the theoretical value of 1 (C = 1 when the male is equally competitive as the wild male) by the one sample *t*-tests (Table 2). At the mating ratios of 1:1:1 (Mean ± 95% CI = 0.71 (-0.56 –-0.03), *t* = -2.253, *df* = 29, *p* = 0.032), 5:1:1 (Mean ± 95% CI = 0.86 (-0.25 –-0.02), *t* = -2.366, *df* = 29, *p* = 0.025) and 10:1:1 (Mean ± 95% CI = 0.70 (-0.46 –-0.15), *t* = -3.948, *df* = 29, *p* = 0.000), the sterile males were significantly less competitive when compared to the wild males. However, when the mating ratio increased to 20:1:1, the sterile males were significantly more competitive when compared to the wild males (Mean ± 95% CI = 1.37 (0.10–0.64), *t* = 2.761, *df* = 29, *p* = 0.010). Therefore, the ratios of 10:1:1 and 20:1:1 should be the optimum release ratios for sterile males, since they could compete with the wild males and could introduce near complete or complete sterility in the wild females respectively.

**Table 2:** Competiveness between irradiated *Wolbachia*-infected males (♂ir-W) vs non-irradiated wild type males (♂nr-w), in order to mate with non-radiated wild type females (♀nr-w) at the ♂ir-W:♂nr-w:♀nr-w ratios of 1:1:1, 5:1:1, 10:1:1 and 20:1:1.

Ratio ♂ir-W:♂nr-w:♀nr-w	No. Replicate	Total eggs (Mean ± SD)	Total hatched eggs (Mean ± SD)	Egg hatch rate (Mean ± SD)	Induce Sterility (IS) (Mean ± 95%CI)	Fried Index (C) (Mean ± 95%CI)
1:1:1	30	1,069 (35.63 ± 9.87)	640 (21.33 ± 9.03)	0.60 (0.59 ± 0.17)	99.34 (-0.73 - -0.59)*	0.71 (-0.56 - -0.03)*
5:1:1	30	1,308 (43.60 ± 1.17)	235 (7.83 ± 2.74)	0.18 (0.18 ± 0.05)	99.80 (-0.22 - -0.18)*	0.86 (-0.25 - -0.02)*
10:1:1	30	1,638 (54.60 ± 8.78)	237 (7.90 ± 3.98)	0.14 (0.15 ± 0.08)	99.83 (-0.20 - -0.13)*	0.70 (-0.46 - -0.15)*
20:1:1	30	1,396 (46.53 ± 17.17)	52 (1.73 ± 0.91)	0.04 (0.0 4± 0.03)	99.95 (-0.06 - -0.04)*	1.37 (0.10–0.64)*

* significant difference at *p* < 0.05

### Assessment of egg hatch rate of wild *Aedes aegypti* females following sterile male release

A total number of 437,980 sterile *Ae*. *aegypti* males, ranging from 9,000 to 25,000 males per week (mean ± SD = 18,245.83 ± 4,972.97), were released over 6 months, or 24-week period, from June to December 2016. This period covered both the rainy season and the cool-dry season, with the rainfall ranging from 0 to 235 mm. The mean egg hatch rates were analyzed in the treatment area, the adjacent area, and the control area in two periods: the first twelve weeks of sterile male release (W1-W12) and the second twelve weeks after the release (W13-W24).

Before the first week of sterile male release (W1), the local public health authority launched a conventional vector control program, including fogging, to aid with the release of the sterile males. This approach was applied in all study areas (i.e., the treatment area, adjacent area, and control area) by public health authorities. As a result, the mosquito abundance declined in all areas where the control operation took place.

The mean egg hatch rate in the treatment area, adjacent area, and control area were then analyzed by using binary logistic regression analysis. The Odds Ratios (OR’s) and 95% confidence intervals (95% CI) were presented; and a *p*-value < 0.05 was considered significant. Results showed that the mean egg hatch rate was lower in the treatment area when compared to the adjacent and control areas, both during the first (W1-W12) and the second twelve weeks (W13-W24). For the first twelve weeks (W1-W12), the mean egg hatch rate was reduced by 45.50% (OR = 0.545, 95%CI = 0.252–1.179, *p* = 0.123) in the treatment area, but it was increased by 1.6 times (OR = 1.620, 95%CI = 0.679–3.862, *p* = 0.277) in the adjacent area when compared to those in the control area. However, no statistical significance was observed (Table 3). On the contrary, the results of the second twelve weeks (W13-W24) showed a huge significant reduction in the mean egg hatch rate, especially in the treatment area (84.00%, OR = 0.160, 95%CI = 0.070–0.368, *p* = 0.000) and the adjacent area (61.20%, OR = 0.388, 95%CI = 0.168–0.897, *p* = 0.027) when compared to those in the control area. This information highlights the importance of a prolonged release of sterile males, since a longer period of release can enhance the reduction in the egg hatch rate.

**Table 3:** Statistical analysis of the egg hatch rate of *Aedes aegypti* during the six-month intervention period in the treatment, adjacent, and control areas located in Plaeng Yao District, Chachoengsao Province, eastern Thailand.

Variables	No. houses	No. ovitraps	No. positive households (Mean ± SD)	Egg hatch rate (Mean ± SD)	Odds Ratio	95% CI	*p*-value
**W1-W12**							
Control area	30	60	22.00 ± 0.43	0.41 ± 0.08	1		
Adjacent area	30	60	24.50 ± 0.39	0.24 ± 0.14	1.620	0.679–3.862	0.277
Control area	30	60	22.00 ± 0.43	0.41 ± 0.08	1		
Treatment area	30	60	18.00 ± 0.50	0.20 ± 0.10	0.545	0.252–1.179	0.123
**W13-W24**							
Control area	30	60	24.50 ± 0.39	0.54 ± 0.11	1		
Adjacent area	30	60	19.00 ± 0.47	0.25 ± 0.16	0.388	0.168–0.897	0.027*
Control area	30	60	24.50 ± 0.39	0.54 ± 0.11	1		
Treatment area	30	60	12.50 ± 0.48	0.18 ± 0.09	0.160	0.070–0.368	0.000*

* significant difference at *p* < 0.05

In conclusion, the sterile male release seemed to show a positive effect in reducing hatched eggs of natural *Ae*. *aegypti* mosquito populations in the treatment area up to 84% when compared to those in the control area after twenty-four weeks (W13-W24). Reduction in egg hatch rate was also observed in the adjacent area that was located 500–800 meters away from the treatment area but with lower percentage (61.20%).

**Fig 2:**
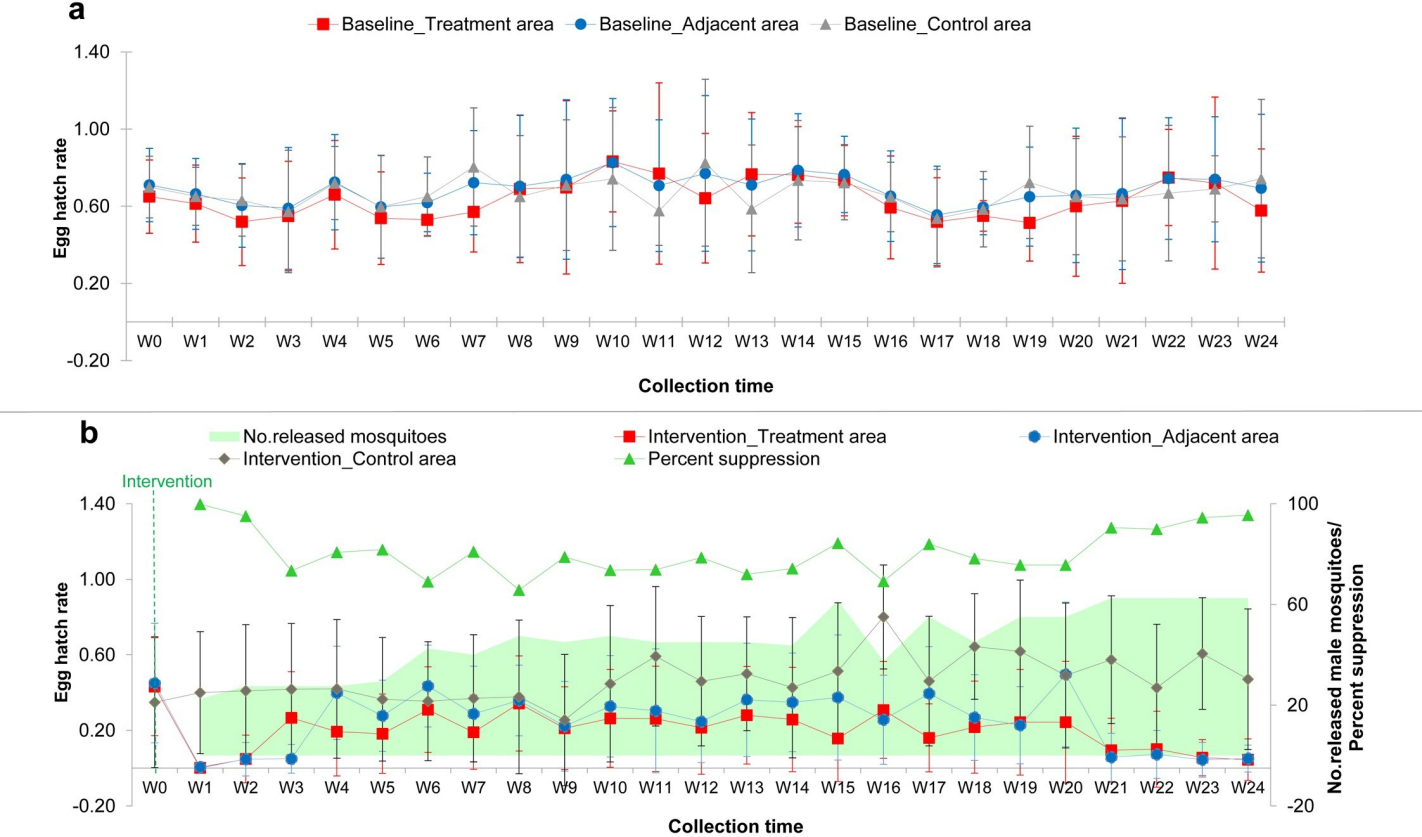
Graph shows the mean egg hatch rate of natural *Aedes aegypti* mosquito populations over time in the treatment, the adjacent and the control areas of the study sites during the baseline (a) and during the intervention (b) periods. Percent suppression efficiency in relation to the number of released sterile males per week is demonstrated in Fig 2B.

Fig 2 shows the mean egg hatch rate of *Ae*. *aegypti* over time in the treatment, adjacent, and control areas of the study sites during baseline (a) and intervention (b) periods. In addition, the suppression efficiency of the release of sterile males during the six-month intervention was evaluated by using the egg hatch rate. Results showed that the suppression efficiency was 80.64 ± 9.36% on average, ranging from 65.73% to 99.86% (Fig 2B).

### Assessment of natural *Aedes aegypti* populations in households following sterile male release

In total, 422 male and 236 female *Ae*. *aegypti* mosquitoes were collected in households by using a combination of the MosHouse sticky traps and MosVac portable vacuum aspirators in the treatment area, adjacent area, and control area in Plaeng Yao District, Chacheongsao Province, eastern Thailand.

Statistical analysis was performed by using the binary logistic regression. The Odds Ratios (OR’s) and 95% confidence intervals (95% CI) were presented; and the *p*-value < 0.05 was considered significant. Results showed that for *Ae*. *aegypti* male mosquitoes, the highest sample collections were from the control area, followed by those from the treatment and adjacent areas, i.e., 193 (32.17 ± 4.07), 137 (22.83 ± 6.55) and 92 (15.33 ± 6.31) respectively (Table 4). When the number of positive households with male collections were considered, it was found that male collections increased by 1.2 times in the treatment area when compared to those in the control area (OR = 1.242, 95%CI = 0.651–2.373, *p* = 0.511); while the numbers of collected males in the adjacent area were 73.70% lower when compared to those in the control area (OR = 0.263, 95%CI = 0.149–0.464, *p* = 0.000) (Table 4). For *Ae*. *aegypti* female mosquitoes, the highest sample collections were also from the control area, followed by those from the adjacent and treatment areas, i.e., 185 (30.83 ± 7.05), 35 (5.83 ± 2.64), and 16 (2.67 ± 1.75) respectively. In conclusion, *Ae*. *aegypti* females, which were the main vector of dengue, were significantly reduced by 97.30% (OR = 0.027, 95%CI = 0.013–0.056, *p* = 0.000) in the treatment area and by 94.40% (OR = 0.056, 95%CI = 0.029–0.108, *p* = 0.0000) in the adjacent areas, when compared to those collected from the control area.

**Table 4:** Comparison of the mean numbers of *Aedes aegypti* males and females collected in the treatment, control, and adjacent areas in Chachoengsao Province, eastern Thailand, during the six-month intervention period.

Variables	No. Houses	No. MosHouse traps	No. positive households (Mean ± SD)	Total mosquitoes (Mean ± SD)	Odds Ratio	95%CI	*p*-value
**Male**							
Control Area	20	20	15.83 ± 1.60	193 (32.17 ± 4.07)	1		
Adjacent Area	20	20	10.00 ± 3.35	92 (15.33 ± 6.31)	0.263	0.149–0.464	0.000*
Control Area	20	20	15.83 ± 1.60	193 (32.17 ± 4.07)	1		
Treatment Area	20	20	16.50 ± 2.88	137 (22.83 ± 6.55)	1.242	0.651–2.373	0.511
**Female**							
Control Area	20	20	17.00 ± 3.22	185 (30.83 ± 7.05)	1		
Adjacent Area	20	20	4.83 ± 1.83	35 (5.83 ± 2.64)	0.056	0.029–0.108	0.000*
Control Area	20	20	17.00 ± 3.22	185 (30.83 ± 7.05)	1		
Treatment Area	20	20	2.67 ± 1.75	16 (2.67 ± 1.75)	0.027	0.013–0.056	0.000*

* significant difference at *p* < 0.05

The lower numbers of females collected in the treatment area when compared to those in the control area could be due to the effect of the sterility that the released sterile males introduced to the wild females. It also reduced *Ae*. *aegypti* mosquito populations in the adjacent area. The higher number of males collected in the treatment area when compared to those in the adjacent area during the intervention could be due to the dispersal of released sterile males into households, since they were also captured and collected by the MosVac portable aspirators and MosHouse sticky traps, together with wild males. Lower male collections in the treatment area when compared to those in the control area indicated that male *Ae*. *aegypti* mosquito populations in the study site were high and continued release of sterile males was necessary in order to compete with wild males.

**Fig 3:**
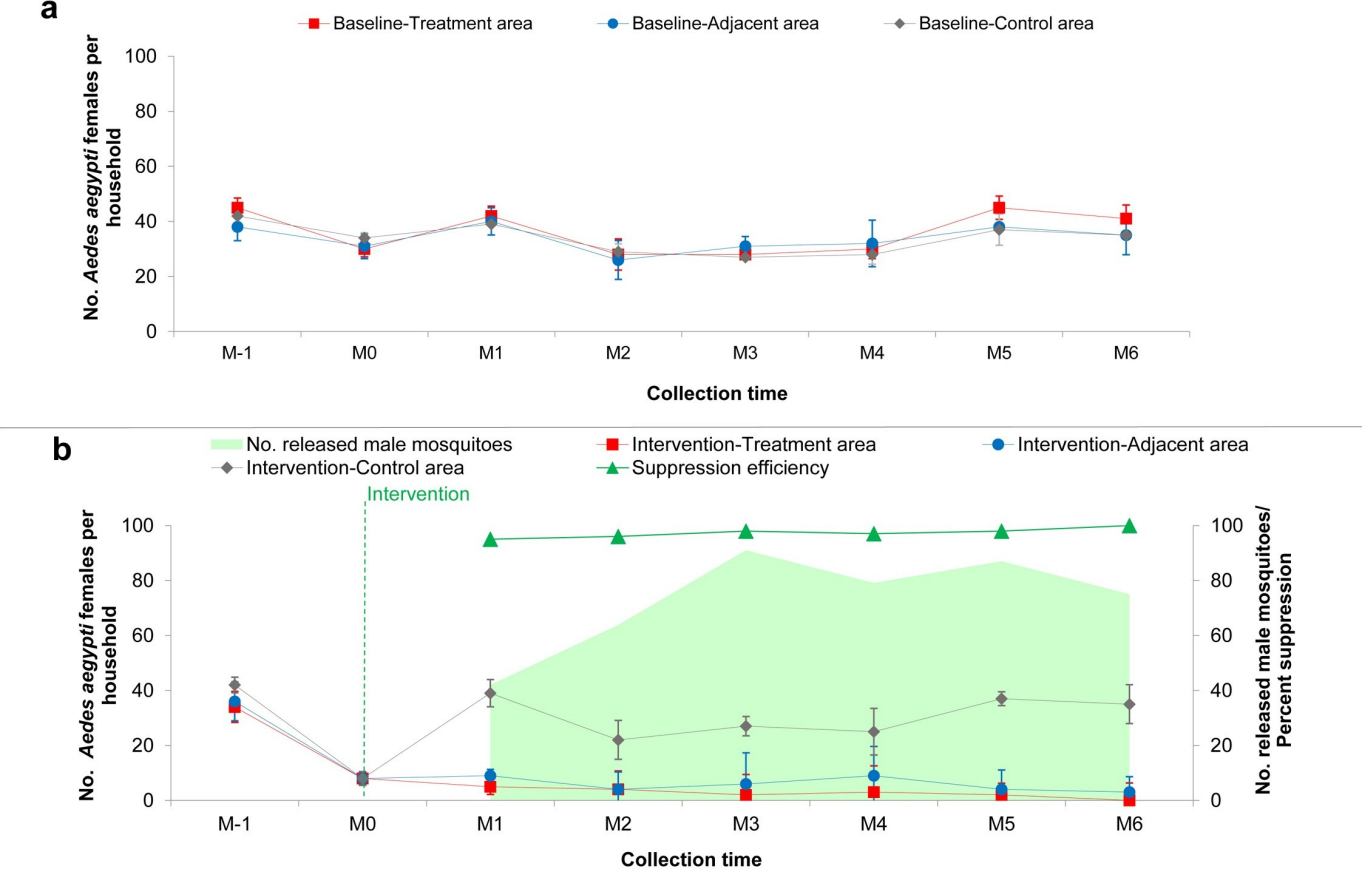
Graph shows the mean number of *Aedes aegypti* females per the study sites during the baseline (a) and during the intervention (b) periods. Percent suppression efficiency in relation to the number of released sterile males per month is demonstrated in Fig 3B.

Fig 3 shows the household abundance of *Ae*. *aegypti* mosquito populations over time in the treatment, adjacent, and control areas of the study sites during the baseline (a) and the intervention (b) periods. During the six-month intervention, the suppression efficiency was evaluated from *Ae*. *aegypti* females collected by using MosHouse and MosVac. Results showed that the suppression efficiency was 97.33 ± 1.75% on average, ranging from 95.00% to 100.00% (Fig 3B).

### Assessment of dispersal and longevity of released sterile males

**Fig 4:**
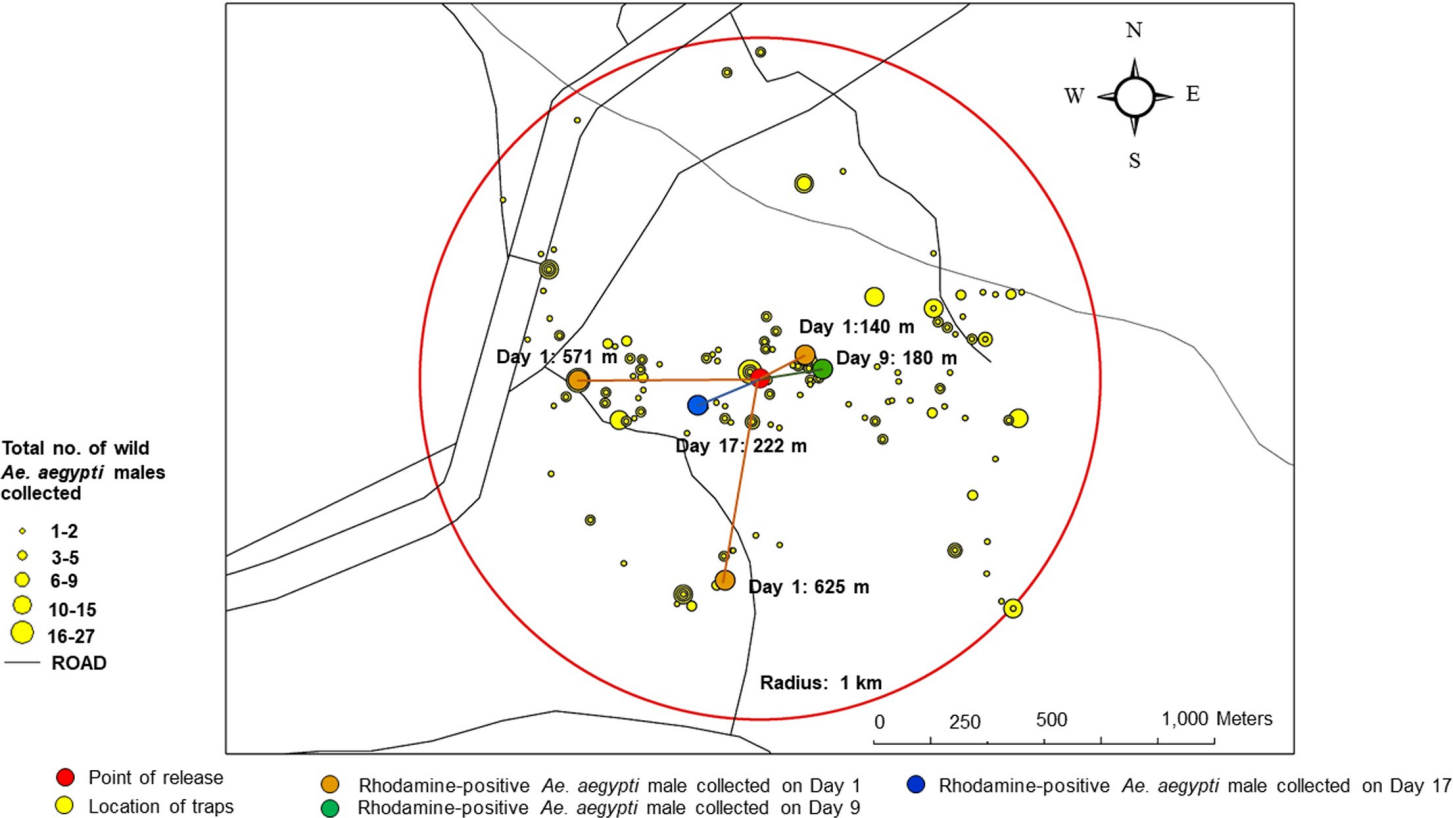
Map shows the dispersal and the longevity of sterile *Aedes aegypti* male mosquitoes collected during the follow-up mark-release-recapture experiment at the study site.

A total number of 786 wild *Ae*. *aegypti* male mosquitoes was collected from the sampling households located within 1-km radius of the study site by MosVac portable vacuum aspirators. Only 5 Rhodamine-marked males were collected back out of 1,400 sterile male mosquitoes released. The rate of recapture was 0.36%. Three males (0.21%) were collected on Day 1 at distances of 140 m, 571 m, and 625 m. The fourth male (0.07%) was collected on Day 9 at a distance of 180 m, and the fifth one (0.07%) was collected on Day 17 at a distance of 222 m from the release point (Fig 4). In conclusion, our experiment showed that the sterile males dispersed at a distance of 379 ± 254.36 meters on average, and as far away as 625 meters. In addition, they lived 5.80 ± 7.02 days on average, and some of them could live up to 17 days in the field situation.

### Assessment of community/public awareness and acceptance of sterile male release

The general public was successfully engaged through several national media reports, national news, and TV and radio programs. A total of 109 media items, including a documentary, international news, national news, national radio, TV shows, and newspaper and online articles, were produced from January 2016 to February 2018 for public education of the sterile male release in Thailand, with a total of 73,098 views and 4,698 shares on social media (Table 5). Online articles published by reliable media publishers seemed to have a higher access in reaching a wide audience, hence the higher number of views and sharing on social media. Moreover, TV shows and a documentary that focused on the topic of the sterile male mosquito gained a lot of public attention. As social media has rapidly developed nowadays, it is an interesting channel to be used to communicate or address important messages to a majority of the public. However, various types of media should be used in order to have effective public education.

**Table 5:** Public awareness, obtained through various media channels, on the pilot project to suppress natural *Aedes aegypti* populations using the SIT/IIT approach in Thailand.

Type of media	No. items	No. views*	No. sharing on social media
Documentary	8	12,409	12
International news	3	N/A	N/A
National news	10	4,272	N/A
TV show	5	7,238	0
Radio	3	N/A	N/A
Newspaper	16	N/A	N/A
Online article	64	49,179	4,686

* Numbers were obtained on November 22, 2018 and did not include the uncountable numbers viewed during live TV broadcasting.

In terms of community acceptance, a total of 173 households in Plaeng Yao District, Chacheongsao Province participated in this study, of which 70 (40.46%), 50 (28.90%), and 53 (30.64%) households were in the treatment area, adjacent area, and control area respectively. In the adjacent area and control area, all participants (100%) showed their acceptance and willingness to participate all along the study. However, for the households in the treatment area, all participants showed full support and participation (100%) at the beginning of the study, but as the release of the sterile males continued consecutively for 24 weeks, some of them (4.29%) preferred to withdraw from the study; still the majority of them continued and participated until the end of the study. Overall, the majority of participants (95.71%) in the treatment area would like to benefit from this study, i.e. larval-pupal survey, adult mosquito removal from households, etc. and were willing to participate if the release of sterile males continued or was implemented in the area. For those who decided not to have the sterile males released in their houses, the owners were elderly, uneducated, could not differentiate male and female mosquitoes in terms of biting ability and were afraid of mosquito bites.

## Discussion

This project demonstrates the first effort to suppress natural populations of *Ae*. *aegypti*, the major vectors of the dengue, chikungunya, and Zika diseases, in a semi-rural village setting in Thailand by releasing sterile males developed from the combined sterile insect technique, using radiation and *Wolbachia*-induced incompatibility (SIT/IIT). Previous studies conducted field trials with sterile males that were produced by either the sterile insect technique (SIT) using radiation or the insect incompatible technique (IIT) through *Wolbachia*-induced cytoplasmic incompatibility, until recently when the combined techniques were applied to *Ae*. *albopictus* (37).

Due to the benefit of being environmental friendly, *Wolbachia* becomes a popular biological control method to combat mosquito vectors and vector-borne diseases. There are two different approaches in using *Wolbachia* in vector-borne disease control, i.e. 1) population suppression that requires males only for release, with the outcome of reducing natural mosquito vector populations and eventually reducing the diseases they transmit; and 2) population replacement where both males and females need to be released, resulting in replacement of natural mosquito vector populations with *Wolbachia*-infected ones. Due to its ability to block viruses, it is expected that the *Wolbachia*-infected mosquitoes no longer will become vectors and transmit vector-borne diseases.

Population suppression through *Wolbachia*-induced CI (or insect incompatible approach) was first deployed in 1967 in Myanmar, in an attempt to suppress *Culex quinquefasciatus* [38]. Later on, an open release of *Wolbachia-*induced incompatible *Ae*. *polynesiensis* males caused significant reduction in the egg hatch rate and a decline of natural populations of this mosquito species in French Polynesia, after a thirty-week open release period [39]. Significant reduction of egg hatch rate in natural populations of *Ae*. *albopictus* was also observed in a field trial in USA, with the introduction of an incompatible strain of *Wolbachia*-infected males; and the method was proposed as bio-pesticide against this mosquito species [40].

In contrast, the approach aiming at population replacement, which releases both male and female *Wolbachia*-infected mosquitoes into the wild, has shown no reduction in the mosquito populations. *Aedes aegypti* trans-infected with *Wolbachia* were first released in Australia to evaluate whether CI induced by *Wolbachia* and its antiviral ability could be applied in vector control. The study showed that *Wolbachia*-infected *Ae*. *aegypti* populations successfully invaded and completely replaced uninfected wild *Ae*. *aegypti* populations [41]. However, the ability of *Wolbachia* to protect these mosquitoes from transmitting diseases to humans has not yet been proven in a field situation. Risk assessment of the *Wolbachia* replacement technology has reported the perception that the threat of dengue fever had been eliminated, resulting in less household mosquito control; and this change of human behavior and attitude toward mosquito control was scored as the highest ranked individual hazard [42].

Sterile Insect Technique (SIT) is considered an environmental friendly method, with no effect on human health, and has been proven to be highly sustainable [43]. This technique has been successfully implemented in many insects of agricultural importance and is considered cost-effective for insect population control [44]. For public health, SIT has been a subject of extended research since the mid-1950s, but it has not yet reached the operational level [45]. However, SIT proved successful in reducing the number of *Ae*. *albopictus* populations up to 68% in pilot field trials in Italy [46–48]. SIT operation for mosquito vectors tends to be long-term, since it does not have immediate effects on the reduction of the targeted species, but it is expected to have an impact in reducing the size of the wild populations in the next generation. In addition, entomological surveillance of the vector populations is essential in order to monitor the impact of the SIT programs [43,45].

Integration of a low irradiation dose with *Wolbachia*-induced cytoplasmic incompatibility (CI) demonstrated that under laboratory conditions, and recently under field conditions, that it could be an efficient strategy in vector control programs targeting population suppression of *Ae*. *albopictus* [35,37,49,50]. The sterilized females that are treated with *Wolbachia* and low-level irradiation cannot produce offspring once they escape from sex sorting and are accidentally released with the sterilized males into the wild. Moreover, the low dose of irradiation has minimal effects on male fitness, suggesting that the combined SIT/IIT could be effective in the field [51]. Our research provides further proof-of-concept that the combined SIT/IIT approach could suppress *Ae*. *aegypti* vector populations. However, due to the incomplete CI of the *Wolbachia*-infected *Ae*. *aegypti* line generated by direct microinjection, we could not maintain male fitness by using low dose radiation as was expected by the combined strategy. Future field trials need to use the *Wolbachia*-infected mosquito lines that could induce complete CI, so that it could take full advantage of the combined SIT/IIT approach. In addition, systematic comparison of the alternative technologies, i.e., SIT, IIT and SIT/IIT, including the cost effectiveness of these approaches, should be conducted.

Our studies showed that under laboratory conditions, the mating ratios from 10:1 to 20:1 were optimum for the release of sterile males, since the sterile males could compete with the wild type males and could introduce nearly complete or complete sterility in the wild type females. Previous studies on *Ae*. *albopictus* suggested a sterile to wild male ratio higher than 5:1 in order to ensure the efficiency of the sterile male release [52]. When the release ratio was higher than 6:1, a reduction of 80% in the fertility of wild females was observed [52]. In our study, significant reduction in the mean number of egg hatch rate and the number of *Ae*. *aegypti* females in the treatment area was observed when compared to the control area, following the release ratios of 10:1 to 20:1. Our results followed the same trend with the studies of Harris et al. (2012) that reported 80% relative reduction in treated versus untreated areas over a 23-week period, with the highest release ratios [51,53] or about a 95% reduction in the local population of *Ae*. *aegypti* and an 81% reduction based on egg trapping according to the studies of Carvalho et al. (2015) [54].

According to the release strategy, it is suggested that the number of released males be adjusted according to environmental or climatic factors. In this study, we increased the number of released males after twelve weeks in order to assure enough numbers of sterile males to compete with the wild ones, as their populations normally increase during the rainy season. Besides, prior to the release of sterile males, appropriate control measures should be applied in order to reduce the initial mosquito populations, so that the efficiency of the sterile male release is increased. In assessing the impact of our sterile male release, it was noticed that the mean egg hatch rate of natural *Ae*. *aegypti* populations in the adjacent area, located approximately 500–800 meters away from the treated area, was significantly decreased when compared to the control area. This effect may have resulted from the sterile males’ ability to disperse at a long distance after being released, as observed by our follow-up mark-release-recapture experiment. Therefore, an adjacent area of at least 500 m is needed between the treatment and the control areas.

In the pilot field trial for sterile male release, *Ae*. *aegypti* mosquito populations in the study areas were monitored through the use of simple adult sticky traps and portable vacuum aspirators. A combination of MosHouse adult sticky traps and MosVac portable vacuum aspirators proved to be an appropriate method to monitor *Ae*. *aegypti* populations in the field during the intervention, since they are low cost, simple, easy to use, and no complicated protocol is needed for operation and maintenance during a long period of entomological surveillance or monitoring. For the MosHouse sticky traps, there is an advantage in the low cost (~ $4.5 per trap) so that surveillance and monitoring could be conducted in wider areas, and the bias in sampling due to localization of *Ae*. *aegypti* populations should be reduced. However, other additional collection methods are encouraged in order to obtain additional entomological data for better evaluation of the impact of the intervention involving sterile male release.

In conclusion, the combined SIT/IIT approach in vector control is an environmental friendly strategy that could be used in combination with other compatible methods in order to reach the highest benefit and successful implementation. In order to obtain a successful program, public/community engagement, involving stakeholders from various sectors, is an essential component. Public and community education, with the key messages, should be appropriate to the target audience. In addition, adjustment of the method of intervention should be conducted in real-time in the field, in order to obtain the most support from the community.

Since there is a significant socioeconomic impact, due to the disease burden caused by the *Ae*. *aegypti* mosquito vector, the proof-of-concept of a combined SIT/IIT approach in suppressing natural populations of this mosquito species should be useful; and it could be an alternative or complementary approach to be applied in several countries facing similar problems. As stated by the Vector Control Advisory Group (VCAG) of WHO, this combined SIT/IIT technology has the potential for long-term control of *Ae*. *aegypti* and *Ae*. *albopictus* mosquitoes. However, further entomological and epidemiological field trials have been strongly recommended to confirm the effectiveness of the technology [55]. Therefore, further research at a larger scale using randomized control trials is needed to determine whether there is a significant impact on the diseases transmitted by *Ae*. *aegypti* mosquitoes after their populations are suppressed by this combined SIT/IIT approach.
